# The Case for Targeted Parenting Interventions with Reference to Intergenerational Transmission of Parenting: Qualitative Evidence from Three Studies of Marginalised Mothers’ and Fathers’ Participation in Parenting Programmes

**DOI:** 10.1080/13575279.2020.1812533

**Published:** 2020-09-25

**Authors:** Katie Buston, Rosaleen O’Brien, Karen Maxwell

**Affiliations:** a MRC/CSO Social and Public Health Sciences Unit, University of Glasgow, Glasgow, UK; b NMAHP Research Unit, Stirling University, Stirling, UK

**Keywords:** Parenting, policy, youth, parenting interventions, intergenerational transmission

## Abstract

The idea that how you were parented is key to how you parent your own children is widely recognisable. It is present in popular cultural references, underpins much policy on families and parenting in the UK, and is supported by a substantive body of academic literature. We explore this concept of intergenerational transmission of parenting, understanding it as the context in which parenting interventions have been implemented. We draw on interview data from three Scottish samples of marginalised parents (*n* = 54) to explore how participants think their own parenting behaviours have been shaped by their experience of being parented and how they talk about participation in a parenting intervention in relation to this. We find that how these parents have been parented is salient in considering their own parenting behaviour, and is a key context for their engagement with the intervention. We make the case for parenting interventions targeted at marginalised parents, arguing that they are acceptable to, and useful for, these parents and may, potentially, be effective in breaking cycles of negative parenting. Policy-makers should not shy away from implementing targeted parenting programmes as part of endeavours to address negative parenting.

## Introduction

The idea that how you were parented is key to how you parent your own children is widely recognisable. It is present in popular cultural references, has underpinned policy on families and parenting in the United Kingdom (UK), and there is a substantive body of academic literature supporting it.

Culturally, the oft-cited line, “They fuck you up, your mum and dad” (Larkin, 1988) encapsulates the concept of (negative) intergenerational transmission of parenting. In the poem *This Be the Verse* the parents (and grandparents before them) are portrayed as detrimentally, and inevitably, shaping what their child becomes, including how s/he will parent her/his own children in a cycle that will go on in perpetuity. Many television shows, a current example *This is Us*, feature flashbacks to the characters’ childhoods. Central to story lines is an exploration of how what the characters have become—including the parents they have become—has been shaped by their own upbringings. Neither does one need to spend long searching parenting sites such as Mumsnet to find threads which discuss the extent to which, and how, one’s own experience of being parented shapes one’s own parenting (e.g. MummyDoIt’s contribution to a thread about how people who experienced “toxic parenting” have parented their own children; she describes how her mother repeated the appalling parenting she herself had experienced). (https://www.mumsnet.com/Talk/relationships/510319-toxic-parents-toxically-parented-people-with-grown-up-children). The deterministic aspect of poor parenting begetting poor parenting is often emphasised in popular culture. There is far less representation of parents who have “escaped their past” and not made the same mistakes their own parents did in relation to mothering/fathering.

For around 50 years in the UK, intergenerational transmission of parenting has also underlain political rhetoric regarding families. Parenting has been repeatedly conceptualised as one of the core mechanisms through which cycles of deprivation—key to understanding health and other inequalities—are perpetuated (Gillies, 2007). From Conservative minister Keith Joseph’s seminal speech on cycles of deprivation in the early 1970s (Denham, 2002; Welshman, 2005, 2014), through to Labour Prime Minister Tony Blair’s Respect Action Plan in the 1990s (Respect Task Force, 2006; Welshman, 2014) and on to the Troubled Families programme as introduced by the Coalition Government (Cameron, 2011; Marjoribanks & Davies, 2016), the focus has been on breaking undesirable historic cycles which have at their centre “poor parenting”. Implicit in these policies is the idea that problem families beget problem families. These problem families are framed as a costly societal problem. In contrast, policy developments in Scotland (see also Leadsom, Field, Burstow, & Lucas, 2014 at Westminister), have emphasised the critical nature of the early years of a child’s life in his or her development. The importance of supporting families in facilitating attachment and parenting skills to bolster positive parent-infant relationships at this key time has been emphasised. It is posited that this will have long lasting implications for health (Scottish Government, 2008). Underlying this is a similar need to break intergenerational cycles (see also Scottish Government, 2018), even though “the problem” in these more recent policy documents is defined much more in terms of improving life for children in the early years and into the future. In more recent years, then, there has been somewhat of a qualitative shift away from blaming particular parents for societal ills towards highlighting that there is a need to provide greater support to particular parents in order to improve outcomes for their children. The concept of intergenerational transmission of parenting has, though, been consistently key to understanding the context in which parenting interventions have been implemented.

Alongside popular and political recognition that parenting behaviours are transmitted from generation to generation, the academic literature on intergenerational transmission of parenting has developed. Work has confirmed, through empirical analysis, that intergenerational transmission of parenting does occur (Belsky, 1978, 1980; Belsky, Conger, & Capaldi, 2009; Belsky, Jaffee, Sligo, Woodward, & Silva, 2005; Chen & Kaplan, 2001; Cicchetti & Rizely, 1981; Erzinger & Steiger, 2014; Jeon & Neppl, 2016; Madden et al., 2015; Spinetta & Rigler, 1972; Sroufe, Egeland, Carlson, & Collins, 2005). There is significant, though modest, continuity in parenting across generations (Belsky et al., 2009; Serbin & Karp, 2003). How, precisely, behaviours are transmitted is less clear (Mileva-Seitz, Bakermans-Kranenburg, & IJzendoorn, 2016). There is most support for the key mechanism being the parent having learnt behaviour from his/her own parents which s/he goes on to replicate on becoming a parent him/herself (Bandura, 1977). This is supplemented by less conscious replication of behaviours stemming from how s/he remembers being parented, which will have been shaped by many other relationships and events in one’s life in the intervening years (Crittenden, 1984; Main, Kaplan, & Cassidy, 1985; Mercer, 2006; Quinton, 1988; Simonton, 1983; van Ijzendoorn, 1992). Current social networks, partner, child’s personality, prevalent social norms, resources such as time, money and education, mental health, and genetic factors also influence parenting behaviours, independent of the influence of parenting received (Bronfenbrenner, 1979; Jack, 2000; Mileva-Seitz et al., 2016; Pederson, 2016). As the field has developed, and it has been established that intergenerational transmission of parenting does occur, there has been a move towards uncovering moderating and mediating factors. These have focused on conditions under which transmission does and does not transpire (Belsky et al., 2009; Madden et al., 2015). Both genetic and environmental factors, and gene-by-environment interactions (Mileva-Seitz et al., 2016), are likely to explain why some parents are more, and others are less, likely to replicate parenting behaviours and styles.

These aspects of popular culture, political debate and policy, and of academic research are the context in which the implementation of parenting programmes in the UK can be better understood. Similar discourses of parenting and related policy solutions have emerged across Europe and the Anglophone world (Dermott & Pomati, 2016; Sihvonen, 2016). Usually there has been some degree of targeting, with shorter term aims around improving health and other outcomes for parent and child dyads, but with, often implicit, longer term intergenerational aims. Examples include the adoption of the Triple P suite of parenting programmes by NHS Greater Glasgow and Clyde and Glasgow City Council in Scotland in 2009. The aim was to embed it as a component of early intervention across the city, delivered throughout the area by a range of statutory and third sector agencies to parents who it was felt could benefit from it (NHS Greater Glasgow and Clyde and Glasgow City Council, 2009). In England, the Parenting Early Intervention Programme (PEIP) provided government funding to all 150 local authorities to deliver evidence-based parenting programmes to those who were identified as being in greatest need of such intervention (Lindsay et al., 2011). Since David Cameron resigned as Prime Minister in 2016 there have been few policy developments in this area emanating from the UK Government. At more local levels, however, implementation of parenting programmes continues. In Scotland, for example, delivery of Family Nurse Partnership (FNP) has been targeted at teenage mothers (Ormiston et al. 2014). Both Triple P and FNP have a fairly extensive international evidence base (see for example Department of Health, 2011; Sanders, Kirby, Tellegen, & Day, 2014; P. Wilson et al., 2012). The latest evaluation of FNP in Scotland outlines its value to clients, service providers and other stakeholders (Scottish Government, 2019), though an evaluation of Triple P in Glasgow city found that levels of mental health problems in preschool children did not improve following its implementation (Marryat, Thompson, & Wilson, 2017). There remains, though, the political will to help fix social problems through such interventions (https://www.gov.scot/policies/maternal-and-child-health/family-nurse-partnership/).

In this article, we present qualitative data from three studies which interviewed 54 largely marginalised parents/parents-to-be or their partners participating in one of three parenting interventions in Scotland, UK. We use the term “marginalised” here to refer to parents who may be considered disadvantaged due to experiencing social inequality, parental stress, and/or reduced capacity for developing a healthy attachment relationship (Henderson et al., 2019; National Institute of Health and Clinical Excellence (NICE), 2010; Scharff-Smith & Gampell, 2011). The focus on marginalised families meets a key gap in a literature which has focused disproportionately on affluent parents from middle class areas who are not experiencing complex and multiple problems as families (see Barlow, Kirkpatrick, Stewart-Brown, & Davis, 2006). It is particularly important to focus on marginalised parents because a stressful environment during pregnancy and beyond, with concomitant mental health problems or addictions, for example, often shapes an extremely difficult child-rearing context (Henderson et al., 2019). Situational factors such as living in poverty, or being incarcerated, can make multiple aspects of parenting highly challenging (Gillies, 2007). Outcomes for children are often disadvantaged (see for example Heinecke Thulstrup & Eklund Karlsson, 2017; Mantymaa, Puura, Luoma, Salmelin, & Tamminen, 2004). We focus on the accounts of the parents participating in the three studies to look first, at how they think their own parenting behaviours have been shaped by their experience of being parented, and second, at how they talk about participation in a parenting intervention in relation to this. We build on their perspectives to make a case for targeted parenting interventions which explicitly tackle intergenerational transmission.

## Methods

Data have been drawn from three studies, all of which have sampled largely marginalised parents/parents-to-be who have participated in a parenting intervention, or whose partner has done so in the case of some of the men. The first author (KB) oversaw each study. Details of the respective study designs and methods, as well as the content of each of the parenting interventions, have been documented in detail elsewhere (Buston, 2018a, 2018b; Henderson et al., 2019; Maxwell, 2018; O’Brien et al., 2019). Each of the studies set out to explore, amongst other areas, the upbringing and current situation of respondents, particularly in relation to parenting; whether the parenting intervention the respondents participated in was acceptable to them; and what they did (and/or did not) value about it. Although each study was framed differently, focused on a different group of parents, and, for the YOI study, the parenting intervention was different, there was much overlap in study aims. This paper brings together the data collected to answer each of primary research questions (see Introduction, above), conjoining the analysis to answer them. Ethical approval was obtained from the University of Glasgow’s College of Social Sciences Ethics Committee (study 1 and study 3), the Scottish Prison Service’s Research Access and Ethics Committee (study 1) and the NHS West of Scotland Research Ethics Committee (study 2).

Study 1 (referred to here as YOI study) was an evaluation of a parenting intervention called *Being a Young Dad*. *Being a Young Dad* was delivered to young fathers incarcerated in a Young Offender Institution (YOI). All six of the young men who participated in one of two deliveries of the programme, and who were still incarcerated a month following completion of the programme, were interviewed. They were asked a series of open ended questions focusing on: their motivation for attending the parenting programme; their views on recruitment to the programme; their participation and engagement with the programme and how they felt about individual sessions; how they thought it had increased their knowledge, changed their attitudes, and changed their behaviour with regard to aspects of parenting their child; and their lives as sons and fathers, as well as their identities as such both within and outwith the prison.

Studies 2 and 3 (referred to here as *THRIVE mother study* and *THRIVE father study*) were components of the Trial of Healthy Relationship Initiatives for the Very Early Years (THRIVE). THRIVE is a randomised controlled trial evaluating the effectiveness of two ante-natal parenting interventions (Enhanced Triple P for Baby (ETPB) and Mellow Bumps (MB) aimed at improving maternal mental health and parenting skills, against care as usual (CAU). Participants were recruited through the Special Needs in Pregnancy (SNIPs) protocol used by National Health Service Greater Glasgow and Clyde, which identified women with, for example, histories of domestic violence, mental illness, substance misuse, being looked after in local authority care, or criminal justice involvement. For the *THRIVE mother study* a sub-sample of 26 mothers and mothers-to-be who were taking part in the wider THRIVE study were interviewed, at 2 time points. The first interview took place after the end of the antenatal parenting intervention, and before the birth of the baby. Open questions were asked about the women’s background, including the nature of their additional health and social care needs; the circumstances surrounding their pregnancy; their experiences of being a parent and being parented, recruitment to the trial, and participating in the intervention; and whether they felt the intervention changed their knowledge, attitudes or behaviour in relation to parenting The second interview took place around six months after the birth of their babies and focused on their lives since the birth, and any sustained benefits or negative effects of their participation in the parenting interventions. For the *THRIVE father study* a sub-sample of 22 partners of the THRIVE mothers were interviewed. A series of open ended questions was asked, with the repertory grid technique also used (Fransella, Bannister, & Bell, 2004; G. B. Wilson, 2008). The men were asked about their current circumstances; the pregnancy; their ideas about what a “good father” is; their relationship with their partner; their own childhood; what they thought “mother” and “father” roles should be; their experience of fathering; and their attitudes towards participation in a parenting intervention. The repertory grids interview focused on fatherhood to elicit comparisons and generate constructs around how their upbringings had shaped their conceptualisations of fatherhood, and their constructions of good fatherhood. Across the three studies data from 54 parents/parents-to-be were analysed.

The authors became interested in the question of intergenerational transmission following their independent initial analyses of the three data-sets. Each of the three datasets was then further interrogated with this question in mind, with related data extracted and further coded, using the five stages outlined in framework approaches: (further) familiarisation, identifying a thematic framework, indexing, charting, and mapping and interpretation (Ritchie & Spencer, 1994). Data across the three studies, as well as within each, were compared and contrasted, and similarities and differences further explored. The authors were mindful that somewhat different questions had been asked in each study so differences in accounts, from study to study, may be a product of this.

There were also differences in the samples. For the *YOI study*, the men were between 16 and 21 years old with correspondingly young (all pre-school age) children. Most had only one child. The samples for the two THRIVE based studies were more heterogeneous in age: the women were between 16 and 42 years old, and the men between 15 and 49. The women were interviewed while pregnant with the THRIVE child, and/or when the THRIVE baby was 4–12 months old, though around a third already had one or more children. All the men were interviewed before the birth of the THRIVE child, though around half already had one or more children. The interviews with all three sets of respondents confirmed the context of their parenting, with multiple, and often extreme, vulnerabilities referred to by most of those interviewed. Although their life course histories were diverse, there were many shared experiences including chronic mental health problems, addiction, and relationship breakdown with significant others. Most were of low socio-economic status.

## Findings

### Experience of being parented as a shaper of own parenting attitudes and behaviours

Nearly all of the incarcerated fathers and around two thirds of both the THRIVE mothers/mothers-to-be and fathers/fathers-to-be talked about having had unhappy and unstable childhoods. Their experiences of being parented were central to this. Childhoods were characterised by parental addictions, neglect, sexual abuse, experiencing/witnessing domestic abuse, lack of love and safe boundaries, periods in the care system, harsh, violent or absent fathers/father figures, and, less often, absence of mother through death or other circumstances. Often individuals had experienced many of these, as one led to another and/ or become intertwined with further adverse experiences. The focus of the men’s accounts tended to be on their fathers, and the women’s on their mothers, though participants did reflect on their parents generally.

### “Good” fathers, “bad” fathers

The men from the YOI *study* and the *THRIVE father study* shared their conceptualisations of “good” and “bad” fathers during the interviews. They expressed a desire to be “good fathers” to their own children. They often contrasted how they hoped to parent their child with how their own fathers had parented them. Many of them reflected that they had not been a high priority in their father’s life, perhaps because he had suffered from addiction problems, for example, or he had just not been there to prioritise the needs of a child. The missing father was a common feature of the men’s accounts. Many of the men talked about a dad who was completely, or often, absent in a physical sense, or who had little or no emotional connection with his child. Nearly all of the men, often in relation to this, said that their child *should* be a priority in their life. Most talked about their role as a father being a chance to do things differently for their own children. Kevin (YOI *study*), for example, talked about the domestic violence he had witnessed on a regular basis growing up. He vowed it would not be something his children would ever see. Dino (*YOI study*) was also adamant that his son’s childhood would be:

Nothing like it [his violent childhood] at all, and I know that for a fact.

Stephen (*YOi study*) talked about how he was seeking help for his addictions while in prison. He was, motivated by a desire not to repeat the patterns of his own childhood with his own children. Evan (*THRIVE father study*) reflected on his own unstable upbringing, saying:

It’s like I just didn’t feel loved. I think personally, because of my past, I think I’ll be an amazing father, just because I don’t want to make the same mistakes or go along the same path as my parents…there’s no doubting that I’d show love. The child would definitely definitely feel love. They would know that they’re loved.

Neil (*THRIVE father study*) also talked in terms of filling deficits he perceived from his childhood:

I never got a lot of loving but I make sure that my girls and boys do get a lot. What I didn’t get

### Being a better mother than their own mother was

The *THRIVE mothers* did not tend to reflect specifically on “good mothering” (perhaps because of the different questions asked). As did the fathers, however, they expressed determination to provide their child/ren with a different and “better” childhood, through different and “better” parenting than they had had. For example, Nelly (*THRIVE mother study*) said:

I’d like to have slightly better relationship with my baby than what I did with my mum when I was younger, cos me and my mum constantly clashed when we were younger and me and my dad was even worse together.

Similarly, Caroline (*THRIVE mother study*) and Leighla (*THRIVE mother study*) explained:

I got away with a lot of things when I was younger…I got away with things that I wouldn’t let [daughter] get away with…Even like homework. Nobody ever done homework with me so that’s one of the things I’ve always, know how like soon as [daughter] comes in from she was like primary one, I’m like “right, homework”, cause like I can recognise the things that I think people should have done with me.

We didn’t have the best upbringing. We actually brought ourselves up. It was, you know, my mum was never there. She actually kicked us out when we were 15…Yeah, I think it makes you, certainly for me, it makes me absolutely determined that, that’s not going to happen. There’s, you know, well I hope not (laughter). I would hate to find myself in kind of like fifteen years’ time, having the same issues or similar issues with mine [unborn daughter]…it’s engrained in my head that I will do everything that I possibly can, to have one of those lovely mother/daughter relationships that people that I know have.

There was a greater sense of understanding apparent in the accounts of the women around *why* they may have been parented negatively. Some, of the mothers recognised the vulnerabilities of their own mothers, in particular. They expressed a desire, and a determination, not to find themselves in the sort of situation where such parenting was inevitable. Billie (*THRIVE mother study*) talks about her mother’s difficult life with a partner (Billie’s dad) who was a problem drug user.

I’m aware that I got my anxieties from my mum. And I don’t want to pass it on to [name of child]. Cut, end it now, with you, with me. So you know, my mum was 22 when she had us, she’d twins at 22, do you know what I mean? Yeah, very young, in my mind, very, very young…in my opinion she made a lot of wrong choices and she knows that. And it did affect, it did affect mine and my sister’s upbringing hugely…My dad was a drug addict and my mum had that for a long time. And stuck by him…But as a mother, I think a lot of the time she made the wrong choices.

Orla (*THRIVE mother study*) talks about her mother’s use of antidepressants:

I went to the doctors about it [anxiety] it and they gave me antidepressants. I took one tablet and just never, ever took them again because I’m like…I’m not turning out like my mum. My mum’s on the antidepressants an’ I’m like I’m not turning out like that. Not a chance…Cos I’m not gonna do what my mum does and just goes like that “just whatever” and just shoves me off…Not a chance…it’s just mum, it’s just generally…she mucked up with myself and my brothers and sister.

### Replicating how they were parented in their own parenting behaviours

Not all of those interviewed, however, talked about wanting to do things differently from their parents. There was a small number of participants who said they parented/intended to parent in similar ways to how they how been parented. Olivia (*THRIVE mother* study) said:

It is actually quite a similar way [that we parent]. Like, my mum and dad have always, kind of, let us, not off the leash, they wouldn’t let us run wild, but they kind of gave us a bit of rein that let us make our own mistakes, to learn from them kind of thing, and I find myself with [name of partner’s daughter] doing the same thing. So, I do find my mum and dad’s parenting quite similar to the way that I do it.

Similarly, Christian (YOI study) explained:

I just need to take more time out for him and give him the time of day because he’s my son. I just hope, obviously, he has a good life, I can save up, take him holidays and trips and that and for him, not just general things that make him happy. Just like my mum and my gran and my papa as well because—just like the way they were, with me kind of thing.

With only a small number of exceptions, then, and across all three samples, how these parents and parents-to-be had been parented themselves was highly salient to them. There was a commonly expressed determination not to parent like they had been parented, and/or for their child not to have the childhood they had had. This was regardless of the parent’s age, or the number of children they had, though those who described having the most adverse childhoods tended to express this most strongly. This need to parent differently to how they were parented was talked about positively. The men and women were generally clear on what aspects they would change, or have changed, including particular parenting strategies. Most were optimistic that they were/ would be different with their children. However, some (especially the men) did express an anxious uncertainty about how to be the kind of parent they wanted to be, as well as uncertainty about how they would learn to be any other kind of parent—and particularly father—than the one they had. Having had a difficult upbringing was clearly identified as a barrier to being able to be the good father some of the men in the *THRIVE fathers study* and the *YOI study* wanted to be. Dylan (*YOI study*), for example, talked about having been sexually abused during his childhood. He revealed that he was concerned that this, and his father’s part in it, would determine his own behaviour as a parent as he did not know any other way to parent:

The way I look at my kids and I think “man, what am I doing?”, because I wasn’t brought up the best so how am I meant to bring them up?

Tyler (*THRIVE father* study) was also uncertain about how he would go about being a father:

I don’t know how I would bring them up because I don’t know. The way I was brought up is when my ma left we basically got told to do what we want. My dad’s only rule was “do what you want”.

### Participation in a parenting intervention in relation to intergenerational transmission of parenting

Respondents across all three studies talked about how the programme in which they had participated had addressed their own childhood experience of being parented. Generally, this was in overarching terms: reporting that the parenting intervention in which they were involved was useful in this respect. However, the reflective component of MB, which gave opportunities to participants to reflect on this primarily through exercises called *My Island* and *Ghosts from the Past*, was identified as particularly helpful (see Buston, O’Brien, Wight, & Henderson, 2019 for a full analysis). Rita (*THRIVE mother study*), for example, said:

I think in pregnancy, it [the reflective work] is so important, because you do start to…you start to look at your own childhood and things. That was what was quite good that we did, you know, starting to think about things that your parents did that you wouldn’t do. Or things that your parents did that you would do. Like, thinking about your own parenting, and how your upbringing…Aye, so there was two coordinators, and the three of us. Four out of five of us [in the group] had fathers with alcohol issues.

Others, across the studies, recognised that parenting interventions might be able to go beyond providing advice, and provide insight into the complex factors that affect their parenting. Evan (*THRIVE father study*), for example, said:

Maybe because of my past, sometimes I’m a bit cold. Or, not cold but sometimes my defence mechanism would shut down, just block things out. So maybe I’m a bit numb to some of her [partner’s] feelings. Even though I know exactly what she goes through because I went through it myself. But sometimes I just shut down from it, because I had to do that as a kid. I think [the intervention] could be helpful because we both have our own mental health issues.

### Attending Being a Young Dad

Amongst the fathers in the *YOI study*, all described *Being a Young Dad* as useful. The aspect most often identified as positive was the opportunity to hear others (facilitators and other participants) talking about being a father. They said this helped them to think of ways to be a different father to their own father. Dylan, for example, referred to abuse in his early childhood and subsequent separation from his brothers when he was taken into care. He talked about the programme filling in some of the gaps he felt he had as a parent because of his lack of positive role models:

You don’t really get a lot of people like that [the programme facilitators] who would talk about a lot of stuff like that, so yeah, it was good. You know what other people do and see what you can do better and that.

Dylan did not appear to have much confidence in his ability to be a good father. On a number of occasions in the class (observed by KB), as well as when interviewed, he expressed uncertainty around what was “normal” behaviour for a father. He wanted to be a normal father but was not sure how.

### Attending THRIVE parenting interventions

The men in the *THRIVE father study* had more mixed views about the overall utility of the parenting intervention that they/their partner took part in. They did, however, point to hearing about how others parented as particularly useful. Tyler, for example, who attended ETPB with his partner, liked that other people in the group had similar parenting frustrations:

I didn’t think I was daft when I was telling people this stupid pram annoys me [getting on and off the bus], but that annoyed them and all.

Tyler talked about this in the context of what he called an unstable upbringing: a childhood of neglect, parental addiction, and with periods in looked after care. He talked about how neither he nor his partner had role models on which to base being a “good parent” (his words). The parenting group was useful in giving him access to ordinary parenting behaviours and feelings. He described feeling “relaxed and comfortable” when he realised he felt the same as others when undertaking mundane parenting tasks.

The value of shared advice and experiences amongst group members, and between the facilitators and the group, was also drawn out by many of the women in the *THRIVE mother study*. This was particularly in relation to their backgrounds, and how/what they wanted to change in terms of parenting their own children. Being able to share reflections with those with similar backgrounds was regarded as valuable. For example, Zoe, who was sexually abused by her father in childhood, said:

the reason that our Triple P group worked really well was the fact that three of us were in similar positions.

Sharing was helpful in terms of it prompting their own further reflection and learning from others. For some of the women it was also valuable in terms of them feeling they had prompted this in others, and helped others learn. This could, potentially, build confidence and raise self-esteem, including amongst women who had very low confidence and selfesteem as a result of their childhood experiences. Orla said:

The other mums give you advice. Because there’s this one girl, this is her first child and she’s having grief with her mum as well. I was like “Hmm, I know exactly where you’re coming from. I can help you with that”…I’ve always thought I was a bad mum, always. Just because that’s just the way my mum basically said, “You’re doing it wrong, you’re doing this wrong”. So, I thought “Well, I’m not doing anything right”, do you know what I mean? Well, its [the group] helped me a lot. I’m more confident.

Participants across the three studies identified various helpful ways in which the parenting interventions addressed experiences of being parented as a shaper for current parenting. It was a key factor facilitating their engagement with the programmes.

## Discussion

We conclude by making the case for parenting interventions which are targeted at marginalised parents. They are acceptable to, and useful for, these parents. They may, potentially, be effective in breaking cycles of negative parenting. We have presented qualitative evidence that for many of these marginalised parents, how they were themselves parented is often highly salient to them as they parent, or prepare to parent, their own child (ren). Accounts illustrate how many had experienced unstable parenting themselves as children. This is often identified as something they do not want for their own children. While some appear confident that their own children will have more stable upbringings, for others there is doubt. The parenting interventions appear to have potential to reduce this doubt and enable them to believe they will be able to parent their child(ren) in more positive ways through: hearing about the experiences of others and accessing role models, realising that others have similar backgrounds and shared current concerns, better understanding and being able to think through past influences on current behaviours, and being more confident, generally, in parenting (see also Buston et al., 2019).

The analysis has strengths and weaknesses. It can be relatively difficult to recruit respondents with low socio-economic status (Boag-Munroe & Evangelou, 2012; Rockliffe, Chorley, & Marlow, 2018; Shaghaghi, Bhopal, & Sheikh, 2011), so this analysis of the accounts of 54 marginalised parents, many of whom were experiencing high levels of socio-economic deprivation (including prior to incarceration), is important. However, comparing data from three different studies, with slightly different foci and sets of questions asked, has already been identified as having limitations. The analysis has been designed to have breadth, across the studies, rather than depth. Many of the themes touched upon here are being analysed separately, study by study, with papers dedicated to these in progress or planned. The aim of the work presented here is instead to analyse the accounts of three distinct groups of marginalised parents who have (or whose partners have) participated in parenting interventions through the lens of interge-nerational transmission of parenting. It should be acknowledged that as all participants had engaged with a parenting intervention to some extent, the sample is skewed. The analysis is novel in being able to assess the accounts of many highly marginalised parents/parents-to-be—including less often studied fathers as well as mothers—who have had involvement with parenting interventions. It is recognised that those who did not choose to take part in the interventions might have found them neither acceptable nor useful.

The paper has focused on the particular cultural context in the UK, with all three studies sited in Scotland. ETPB (Nowak & Heinrichs, 2008; Sanders, Markie-Dadds, Tully, & Bor, 2000), MB (Breustedt & Puckering, 2013; Puckering et al., 2011) and *Being a Young Dad* (Butler, Hayes, Devaney, & Percy, 2015) have been delivered across the United Kingdom, and Triple P and MB have been delivered internationally There is no reason to suppose that acceptability, and the utility of these interventions via the suggested mechanisms by which intergenerational transmission are potentially addressed, would not be similar in different cultural contexts. Findings will therefore be relevant to broader international debates around parenting, interventions, and family policy.

Concerns have been raised about the focus on parenting as a designated area of policy intervention. The suggestion that parents need help, via such programmes, has been questioned. Parenting interventions have even been deemed to be a means to control and regulate the conduct of parents, particularly when targeted at working class parents (Clarke, 2006; Edwards & Gillies, 2004; Gillies, 2005). The roll out of targeted interventions has been criticised as being driven by a moral agenda, imposing parenting interventions on vulnerable parents in inappropriate and unacceptable ways (Gillies, 2008, 2012). This was not the picture painted by this analysis. Parenting interventions such as MB, ETPB and *Being a Young Dad* were not unacceptable. Indeed, their potential utility was recognised by participants in intergenerational terms. The data suggest that there are mechanisms through which positive changes in parenting behaviours could, potentially, be effected.

We recognise, however, that there is still a danger of equating poor (negative) parenting with poor (not affluent) parents (Dermott & Pomati, 2016). What is highlighted by the analysis here is the prevalence and depth of instability in the childhoods of these parents. Their own experiences of being parented are rarely positive. Most were not living in affluent circumstance, then or now. However, such childhoods have also been experienced by more affluent parents, and there is no reason to suppose that their experience of these parenting interventions would not be similar. This is where moral panics in the media around “problem families” have not been helpful. However, there did not appear to be a stigma attached to participation in parenting programmes amongst the parents interviewed here.

The results presented in this paper suggest that the marginalised parents who took part in three parenting interventions had agency in the decision to take part. These parenting programmes were not only acceptable to them generally, but were deemed to be worthwhile. This included in helping them better understand and address some of the issues they felt stem from their own childhood experiences of being parented, and which may facilitate them in breaking some of the intergenerational cycles of parenting that they wish to, and intend to, break. Most of the parents studied here were well able to identify how they wished to parent, and some of the barriers they faced, with the three interventions they had participated in apparently well aligned to their needs. Given the acceptability of the intervention to these parents who had already chosen to participate, greater efforts should be directed towards recruitment of a wider range of marginalised parents —mothers and, perhaps particularly, fathers—who are considered “hard to reach” (Barlow et al., 2006). Future research should focus on better understanding whether there are particular marginalised parents for whom parenting interventions are not acceptable. Adaptations can be made, if appropriate, to meet the needs of these parents. Such targeted parenting interventions could contribute to supportive family policy for marginalised parents. We acknowledge, however, that there are likely to be more important components of such policy, not least structural changes facilitating income redistribution.

## References

[R1] Bandura A (1977). Social learning theory.

[R2] Barlow J, Kirkpatrick S, Stewart-Brown S, Davis H (2006). Hard-to-reach or out-of-reach? Reasons why women refuse to take part in early intervention. Children and Society.

[R3] Belsky J (1978). Three theoretical models of child abuse: A critical review. International Journal of Child Abuse and Neglect.

[R4] Belsky J (1980). Child maltreatment: An ecological integration. American Psychologist.

[R5] Belsky J, Conger R, Capaldi DM (2009). The intergenerational transmission of parenting: Introduction to the special section. Developmental Psychology.

[R6] Belsky J, Jaffee S, Sligo J, Woodward L, Silva P (2005). Intergeneration trasmission of warm-sensitive-stimulating parenting: A prospective study of mothers and fathers of 3 year old. Child Development.

[R7] Boag-Munroe G, Evangelou M (2012). From hard to reach to how to reach: A systematic review of the literature on hard-to-reach families. Research Papers in Education.

[R8] Breustedt S, Puckering C (2013). A qualitative evaluation of women’s experiences of the Mellow Bumps antenatal intervention. British Journal of Midwifery.

[R9] Bronfenbrenner U (1979). The ecology of human development.

[R10] Buston K, Maycock M, Hunt K (2018a). New perspectives on prison masculnities.

[R11] Buston K (2018b). Recruiting, retaining and engaging men in social interventions: Lessons for implementation focusing on a prison based parenting intervention for young incarcerated fathers. Child Care in Practice.

[R12] Buston K, O’Brien R, Wight D, Henderson M (2019). The reflective component of the Mellow Bumps parenting intervention: Implementation, engagement and mechanisms of change. Plos One.

[R13] Butler M, Hayes D, Devaney J, Percy A (2015). Strengtheing family relations? Review of the families matter programme at Maghaberry prison.

[R14] Cameron D (2011). Troubled families speech.

[R15] Chen Z, Kaplan H (2001). Intergenerational transmission of constructive parenting. Journal of Marriage and the Family.

[R16] Cicchetti D, Rizely R (1981). Developmental perspectives on the etiology, intergenerational transmission and sequelae of child maltreatment: Closing comments for the special section. New Directions for Child Development.

[R17] Clarke K (2006). Childhood, parenting and early intervention: A critical examination of the sure start national programme. Critical Social Policy.

[R18] Crittenden PM (1984). Sibling interaction. Evidence of a generational effect in maltreating infants. Child Abuse and Neglect.

[R19] Denham A (2002). From the ‘cycle of enrichment’ to the ‘cycle of deprivation’: Sir Keith Joseph, ‘problem families’ and the transmission of disadvantage. Benefits.

[R20] Department of Health (2011). FNP evidence summary leaflet.

[R21] Dermott E, Pomati M (2016). ‘Good’ parenting practices: How important are poverty, education and time pressure. Sociology.

[R22] Edwards R, Gillies V (2004). Support in parenting: Values and consensus concerning who to turn to. Journal of Social Policy.

[R23] Erzinger AB, Steiger AE (2014). Intergenerational transmission of maternal and paternal parenting beliefs: The moderating role of interaction quality. European Journal of Developmental Psychology.

[R24] Fransella F, Bannister D, Bell R (2004). A manual for repertory grid technique.

[R25] Gillies V (2005). Meeting parents’ needs? Discourses of ‘support’ and ‘inclusion’ in family policy. Critical Social Policy.

[R26] Gillies V (2007). Marginalised mothers: Exploring working-class experiences of parenting.

[R27] Gillies V (2008). Childrearing, class and the new politics of parenting. Sociology Compass.

[R28] Gillies V, Richter M, Andresen S (2012). The politicization of parenthood: Shifting private and public responsibilities in education and child rearing.

[R29] Heinecke Thulstrup S, Eklund Karlsson L (2017). Children of imprisoned parents and their coping strategies: A systematic review. Societies.

[R30] Henderson M, Wittkowski A, McIntosh E, McConnachie A, Buston K, Wilson P, THRIVE Trial Research Team (2019). Trial of healthy relationship Initiatives for the very early-years (THRIVE) evaluating Enhanced Triple P for Baby Mellow Bumps for those with additional social and care needs during pregnancy and their infants who are at higher risk of maltreatment: Study protocol for a randomised controlled trial. Trials.

[R31] Jack G (2000). Ecological influences on parenting and child development. British Journal of Social Work.

[R32] Jeon S, Neppl TK (2016). Intergenerational continuity in economic hardship, parental positivity, and positive parenting: The association with child behaviour. Journal of Family Psychology.

[R33] Larkin P (1988). Collected poems.

[R34] Leadsom A, Field F, Burstow P, Lucas C (2014). The 1001 critical days: The importance of conception to age two period.

[R35] Lindsay G, Strand S, Band S, Davis H, Conlon G, Barlow J, Evans R (2011). Parenting early intervention programme evaluation.

[R36] Madden V, Domoney J, Aumayer K, Sethna V, Iles J, Hubbard I, Ramchandani P (2015). Intergenerational transmission of parenting: Findings from a UK longitudinal study. European Journal of Public Health.

[R37] Main M, Kaplan N, Cassidy J (1985). Security in infancy, childhood and adulthood: A move to the level of representations. Monographs of the society for research in child development.

[R38] Mantymaa M, Puura K, Luoma I, Salmelin RK, Tamminen T (2004). Early mother-infant interaction, parental mental health and symptoms of behavioral and emotional problems in toddlers. Infant Behavior and Development.

[R39] Marjoribanks D, Davies K (2016). Family policy: An integrated approach?. People, Place and Policy.

[R40] Marryat L, Thompson L, Wilson P (2017). No evidence of whole population mental health impact of the triple P parenting programme: Findings from a routine dataset. BMC Pediatrics.

[R41] Maxwell K (2018). Fatherhood in the context of social disadvantage: Constructions of fatherhood and attitudes towards parenting interventions of disadvantaged men in Scotland.

[R42] Mercer J (2006). Understanding attachment: Parenting, child care, and emotional development.

[R43] Mileva-Seitz VR, Bakermans-Kranenburg MJ, IJzendoorn MHV (2016). Genetic mechanisms of parenting. Hormones and Behavior.

[R44] National Institute of Health and Clinical Excellence (NICE) (2010). Pregnancy and complex social factors: A model for service provision for pregnant women with complex social factors.

[R45] NHS Greater Glasgow and Clyde and Glasgow City Council (2009). Parenting support framework.

[R46] Nowak C, Heinrichs N (2008). A comprehensive meta-analysis of triple P-positive parenting program using hierarchical linear modelling: Effectiveness and moderating variables. Clinical Child and Family Psychology Review.

[R47] O’Brien R, Buston K, Wight D, McGee E, White J, Henderson M (2019). A realist process evaluation of Enhanced Triple P for Baby and Mellow Bumps, within a trial of healthy relationship Initiatives for the very early years (THRIVE): Study protocol. Trials.

[R48] Ormiston R, McConville S, Gordon J (2014). Evaluation of the family nurse partnership programme in NHS Lothian, Scotland: Summary of key learning and implications.

[R49] Pederson S (2016). The good, the bad and the ‘good enough’ mother on the UK parenting forum Mumsnet. Women’s Studies International Forum.

[R50] Puckering C, Connolly B, Werner C, Toms-Whittle L, Thompson L, Lennox J, Minnis H (2011). Rebuilding relationships: A pilot study of the effectiveness of the Mellow Parenting Programme for children with reactive attachment disorder. Clinical Child Psychology and Psychiatry.

[R51] Quinton D, Rutter M (1988). Studies of psychological risk: The power of longitudinal data.

[R52] Respect Task Force (2006). Respect action plan.

[R53] Ritchie J, Spencer L, Bryman A, Burgess R (1994). Analyzing qualitative data.

[R54] Rockliffe L, Chorley AJ, Marlow LAV (2018). It’s hard to reach the “hard-to-reach”: The challenges of recruiting people who do not access preventive healthcare services into interview studies. International Journal of Qualitative Studies on Health and Wellbeing.

[R55] Sanders MR, Kirby JN, Tellegen CL, Day JJ (2014). The triple P-positive parenting program: A systematic review and meta-analysis of a multi-level system of parenting support. Clinical Psychological Review.

[R56] Sanders MR, Markie-Dadds C, Tully LA, Bor W (2000). The triple P-positive parenting program: A comparison of enhanced, standard, and self-directed behavioral family intervention for parents of children with early onset conduct problems. Journal of Consulting and Clinical Psychology.

[R57] Scharff-Smith P, Gampell L (2011). Children of imprisoned parents.

[R58] Scottish Government (2008). The early years framework.

[R59] Scottish Government (2018). Every child, every chance – The tackling child poverty delivery plan 2018-22.

[R60] Scottish Government (2019). Family nurse partnership in Scotland: Revaluation report.

[R61] Serbin L, Karp J (2003). Intergenerational studies of parenting and the transfer of risk from parent to child. Current Directions in Psychological Science.

[R62] Shaghaghi A, Bhopal RS, Sheikh A (2011). Approaches to recruiting ‘hard-to-reach’ populations into research: A review of the literature. Health Promotion Perspectives.

[R63] Sihvonen E (2016). Early interventionist parenting support: The case study of Finland. Families, Relationships and Societies.

[R64] Simonton DK (1983). Intergenerational transfer of individual differences in hereditary monarchs: Genetic, role-modelling, cohort, or socio-cultural effects. Journal of Personality and Social Psychology.

[R65] Spinetta J, Rigler D (1972). The child-abusing parent: A psychological review. Psychological Bulletin.

[R66] Sroufe L, Egeland BR, Carlson EA, Collins W (2005). The development of the person: The Minnesota study of risk and adaptation from birth to adulthood.

[R67] van Ijzendoorn MH (1992). Intergenerational transmission of parenting: A review of studies in nonclinical populations. Developmental Review.

[R68] Welshman J (2005). Ideology, social science, and public policy: The debate over transmitted deprivation. Twentieth Century British History.

[R69] Welshman J (2014). From transmitted deprivation to social exclusion.

[R70] Wilson GB (2008). Conflict and co-parenting: The constructs of nonresident fathers. Family Court Review.

[R71] Wilson P, Rush R, Hussey S, Puckering C, Sim F, Allely C, Gillberg C (2012). How evidence-based is an ‘evidence-based parenting program’? A PRISMA systematic review and metaanalysis of triple P. BMC Medicine.

